# Prevalence and risk factors associated with *Campylobacter* spp. occurrence in healthy dogs visiting four rural community veterinary clinics in South Africa

**DOI:** 10.4102/ojvr.v86i1.1673

**Published:** 2019-05-28

**Authors:** Musafiri Karama, Beniamino T. Cenci-Goga, Alice Prosperi, Eric Etter, Saeed El-Ashram, Cheryl McCrindle, Jackson N. Ombui, Alan Kalake

**Affiliations:** 1 Department of Paraclinical Sciences, Faculty of Veterinary Science, University of Pretoria, Onderstepoort, South Africa; 2 Department of Veterinary Medicine, Laboratorio di Ispezione degli alimenti di origine animale, University of Perugia, Perugia, Italy; 3 Experimental Zooprofilattico Institute of Lombardy and Emilia-Romagna ‘Bruno Ubertini’, Brescia, Italy; 4 Department of Veterinary Medical Sciences, University of Bologna, Bologna, Italy; 5 Department of Production Animal Studies, Faculty of Veterinary Science, University of Pretoria, Onderstepoort, South Africa; 6 Centre de Coopération Internationale en Recherche Agronomique pour le Développement-INRA, UMR ASTRE Baillarguet International Campus, University of Montpellier, Montpellier, France; 7 College of Life Science and Engineering, Foshan University, Foshan, China; 8 Faculty of Science, Kafr ElSheikh University, Kafr El Sheikh, Egypt; 9 Department of Agriculture and Animal Health, University of South Africa, Johannesburg, South Africa; 10 Department of Public Health, Pharmacology and Toxicology, College of Agriculture and Veterinary Sciences, University of Nairobi, Nairobi, Kenya; 11 Gauteng Department of Agriculture and Rural Development, Gauteng, Johannesburg, South Africa

**Keywords:** dogs, *Campylobacter* spp., *C. jejuni*, *C. coli*, *C. upsaliensis*, risk factors, South Africa

## Abstract

Reports on the occurrence of *Campylobacter* spp. in dogs in South Africa are non-existent. This study investigated the prevalence of *Campylobacter* spp. in 481 dogs visiting four rural community veterinary clinics in South Africa. Dogs were screened for *Campylobacter* spp. by culture and polymerase chain reaction (PCR), and logistic regression analysis was performed to assess the association between sex, clinic, breed and age and the occurrence of *Campylobacter* spp. in dogs. The prevalence of *Campylobacter* spp. was 41.50% (95% confidence interval [CI], 37.39% – 46.04%). *Campylobacter jejuni, C. upsaliensis* and *C. coli* were detected in 29.31% (95% CI, 25.42% – 33.54%), 13.10% (95% CI, 10.37% – 16.42%) and 5.41% (95% CI, 3.71% – 7.82%) of dogs, respectively. Dogs carrying more than one species of *Campylobacter* spp. accounted for 6.23% (95% CI, 4.40% – 8.78%). *Campylobacter upsaliensis* and *C. jejuni* were detected in 3.74% (95% CI, 2.37% – 5.86%), whereas *C. coli* and *C. jejuni* were found in 2.49% (95% CI, 1.42% – 4.34%) of dogs. Age and clinic were the risk factors significantly associated with *Campylobacter* spp. occurrence, while age, breed and clinic were predictors of *C. jejuni* carriage. Furthermore, age was the only risk factor associated with a higher likelihood of carrying *C. upsaliensis*. The prevalence of *Campylobacter* spp. *C. jejuni* and *C. upsaliensis* increased significantly as dogs grew older. In addition, the odds of carrying *Campylobacter* spp. were higher in the Staffordshire bull terrier breed compared to crossbreed dogs. In conclusion, this study shows that dogs visiting rural community veterinary clinics in South Africa are reservoirs of *Campylobacter* spp. and may be potential sources of *Campylobacter* spp. for humans living in close proximity of the dog populations under study.

## Introduction

*Campylobacter* spp. are the leading cause of bacterial diarrhoeal diseases globally. Around 400–500 million cases of *Campylobacter* infections occur each year globally (Friedman et al. 2000). Although most human cases of campylobacteriosis are foodborne or waterborne (Jacobs-Reitsma, Lyhs & Wagenaar 2008), a number of studies have shown that contact with dogs is a risk factor for human campylobacteriosis (Couturier, Hale & Couturier 2012; Damborg et al. 2016; Man 2011; Mughini Gras et al. 2012; Neimann et al. 2003; Rossi et al. 2008; Tenkate & Stafford 2001). Manifestations of *Campylobacter* infections in humans include mild watery to severe bloody diarrhoea, nausea and vomiting and in some cases life-threatening complications such as Guillain-Barré syndrome or its variant, Miller-Fisher syndrome (Jacobs et al. 2008). In immunocompromised patients, *Campylobacter jejuni* is an important cause of severe bacteraemia, which may lead to death (Tee & Mijch 1998).

Dogs are considered asymptomatic carriers of *Campylobacter* spp. (Acke et al. 2009; Carbonero et al. 2012; Hald & Madsen 1997). A number of studies have reported *Campylobacter* spp. carriage rates ranging from 2.7% to 100% in dogs (Chaban, Ngeleka & Hill 2010; Hald et al. 2004; Tsai et al. 2007). Furthermore, clinical cases in dogs with *Campylobacter* spp. as a primary or secondary cause of diarrhoea have also been reported (Burnens, Angéloz-Wick & Nicolet 1992; McOrist & Browning 1982). Healthy and diarrhoeic dogs may harbour one or more *Campylobacter* species including *C. jejuni, C. coli* and *C. upsaliensis* (Carbonero et al. 2012; Chaban et al. 2010; Giacomelli et al. 2015; Holmberg et al. 2015; Parsons et al. 2011). Both *C. jejuni* and *C. upsaliensis* have been implicated in dog-associated C*ampylobacter* spp. infections in humans (Bourke, Chan & Sherman 1998; Couturier et al. 2012; Nachamkin, Allos & Ho 1998). Zoonotic transmission of *Campylobacter* spp. from dogs occurs through direct contact with infected dogs or dog faeces (Damborg et al. 2016; Mughini Gras et al. 2012). There are suggestions that contact with dogs may be associated with up to 6% of human campylobacteriosis cases (Rossi et al. 2008; Tenkate & Stafford 2001).

Although dogs are considered an important reservoir of *Campylobacter* spp. (Hald & Madsen 1997; Hald et al. 2004), current data on the prevalence of *Campylobacter* spp. in dogs in South Africa and on the African continent are lacking. Therefore, the aim of this study was to investigate the prevalence and risk factors associated with *Campylobacter* spp. occurrence in healthy dogs visiting four rural community veterinary clinics in South Africa.

## Materials and methods

### Study design, area and population

**FIGURE 1:**
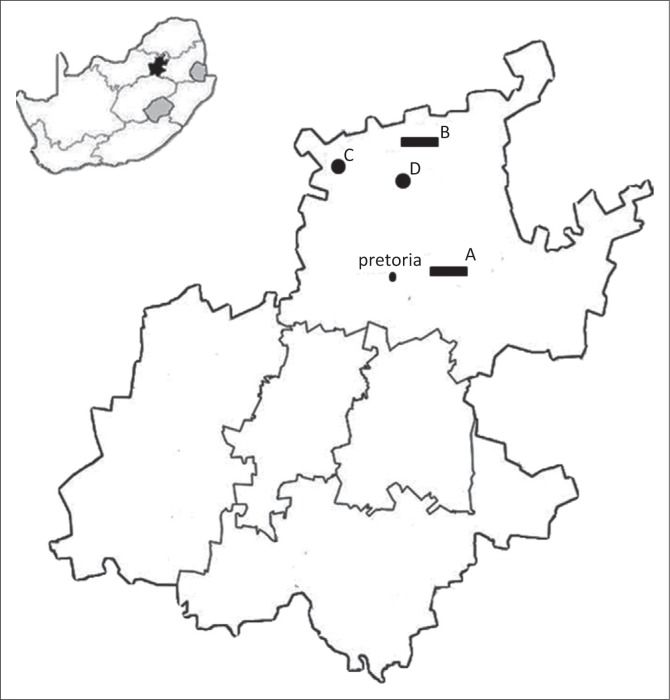
A map of Gauteng Province showing the four clinics and/or sites where the samples were collected.

This cross-sectional study was conducted at four rural community veterinary clinics located in Gauteng Province, South Africa (see Figure 1). A total of 481 dogs were screened for *Campylobacter* spp. including *C. jejuni, C. coli* and *C. upsaliensis*. Each dog owner had an identity card on which the following variables were recorded: name of the dog and owner, vaccination status, sex, date of birth and breed. Faecal swabs were obtained from all dogs that visited a particular clinic on the sampling day. This study was approved by the Animal Ethics Committee of the University of Pretoria (V056-15).

### Sample collection and *Campylobacter* spp. culture methods

Sterile swabs were used to collect faecal samples from dogs during routine vaccination and deworming campaigns. Each clinic was visited once, and one sample was obtained from each animal. For culture and isolation of *Campylobacter* spp., swabs were spread-plated on Campy CVA agar (Brucella agar containing 5% defibrinated sheep blood supplemented with 20 mg of cefoperazone, 10 mg of vancomycin and 2 mg of amphotericin B) (Becton, Dickinson and Company, Sparks, MD, United States [US]). The inoculated plates were incubated at 37 °C for 48–72 hours in tightly sealed anaerobic system containers in which GasPak^TM^ EZ Campy System sachets (Becton, Dickinson and Company, Sparks, MD, US) were placed to create a microaerophilic atmosphere (approximately 6% – 16% oxygen and 2% – 10% carbon dioxide). We used 37 °C to incubate *Campylobacter* spp. instead of the generally used 42 °C to mimic the ‘natural’ growth environmental temperature of *Campylobacter* spp. in the gastrointestinal tract of dogs. Furthermore, 37 °C was used to favour the growth of *C. upsaliensis*, which grows optimally at 37 °C (Lastovica & Le Roux 2001).

### DNA extraction

Briefly, a sterile inoculating loop was used to harvest colony sweeps from all Campy CVA plates that showed growth after 48 h – 72 h. A loop-full of colony sweeps was suspended in a 1.5 mL Eppendorf tube containing 1 mL of FA buffer (Becton, Dickinson and Company). Bacterial suspensions were mixed and washed by vortexing, followed by centrifugation (15 000 g) for 5 minutes. After the first wash and centrifugation cycle, the supernatant was discarded and the bacterial pellet was resuspended in FA buffer (Becton, Dickinson and Company). Two additional washes and centrifugation cycles were carried out, after which the pellet was suspended in 500 *µ*L of sterile water, vortexed and the homogeneous cell suspension was boiled to 100 °C for 15 min, then stored at -20 °C for further processing.

### *Campylobacter* spp. screening

A *Campylobacter* spp. specific multiplex polymerase chain reaction (PCR) protocol (Forbes & Horne 2009) was used to screen DNA for *Campylobacter* spp. (*C. jejuni, C. coli* and *C. upsaliensis*). Primers lpxA*C. jejuni*-ACAACTTGGTGACGATGTTGTA (Klena et al. 2004), lpxA*C. coli* GATAGTAGACAAATAAGAGAGAATMAG (Forbes & Horne 2009) and lpxA*C. upsaliensis*-AAGTCGTATATTTTCYTACGCTTGTGTG (Klena et al. 2004) were used as forward primers. Primers lpxA-R1-CAATCATGTGCGATATGACAATAYGCCAT, lpxA-R2-CAATCATGA-GCAATATGACAATAAGCCAT and lpxAR KK2m CAATCATGDGCDATATGASAATAHGCCAT were used as reverse primers for *C. jejuni, C. coli* and *C. upsaliensis* (Klena et al. 2004; Forbes & Horne 2009). Each PCR reaction (25 *µ*L) contained 2.5 *µ*L of 10X Thermopol reaction buffer, 2.0 *µ*L of 2.5 mM deoxyribonucleotides triphosphate (dNTPs), 0.25 *µ*L of 100 mM MgCl2, 1.25 *µ*L of 0.5 *µ*M of each forward primer and 1.25 *µ*L of 0.25 *µ*M of each reverse primer in a 50:50 mixture, 1U of Taq DNA Polymerase (New England BioLabs^®^ Inc., Ipswich, MA, US) and 5 *µ*L of DNA template. Sterile water was used to top up the reaction volume to 25 *µ*L. *Campylobacter jejuni* ATCC 33560, *C. coli* derived from ATCC 33559 (Microbiologics, St Cloud, MN, US) and an in-house dog *C. upsaliensis* isolate were used as positive controls, and sterile water was the negative control. All PCR reagents were supplied by New England BioLabs, except for the primers, which were supplied by Inqaba Biotec (Inqaba Biotec, Pretoria, South Africa) or Integrated DNA Technologies (IDT, San Diego, CA, US). Polymerase chain reactions were performed in a C1000 Touch^TM^ (Bio-Rad, Hercules, CA, US) or a Veriti™ (Applied Biosystems^®^, Foster City, CA, US) thermal cycler. Amplified DNA was electrophoresed in 2.5% (w/v) agarose gels in tris-acetate-ethylenediaminetetraacetic acid buffer. Gels were stained with ethidium bromide (0.05 mg/*µ*L), and amplicons were visualised under ultraviolet light in a Gel Doc system (Bio-Rad, Hercules, CA, US).

### *Campylobacter* speciation

Colony sweeps were obtained from all Campy CVA plates that were positive for *Campylobacter* spp. on PCR screening, streaked on horse blood agar and incubated at 37 °C for 48 h – 72 h to obtain single colonies. Three suspect *Campylobacter* spp. single colonies were taken from each horse blood agar plate with a sterile plastic inoculating loop or swab, spread-plated separately on horse blood agar plates and incubated at 37 °C for 48 h – 72 h to multiply and purify the single colonies. After incubation, pure single colony bacterial sweeps were harvested using a sterile plastic inoculating loop or swab, and the bacterial cells were suspended in 1.5 mL FA buffer in an Eppendorf tube. DNA was extracted from the single colony sweeps by the boiling method and screened for *C. jejuni, C. coli* and *C. upsaliensis* using the aforementioned primers and multiplex PCR protocol (Forbes & Horne 2009; Klena et al. 2004). Single colony isolates that were confirmed as *C. jejuni, C. coli* or *C. upsaliensis* on PCR were stored at -80 °C in cryovials containing a sterile freezing mixture (70% Brucella broth and 30% glycerol).

### Statistical analysis

All statistical analyses were performed using the ‘base’, ‘epiDisplay’ and ‘aod’ packages of the R software version 3.3.3 (R Foundation for Statistical Computing 2017, Vienna, Austria, http://www.R-project.org/). The prevalence of *Campylobacter* spp., *C. jejuni, C. coli* and *C. upsaliensis*, were computed on 481 dogs using a general linear model considering the error distribution as binomial and the link function ‘logit’ (logistic regression). The following risk factors were tested: sex, breed, clinic, age and number of vaccinations. As a categorical variable, ‘breed’ included crossbreed, molosser, Staffordshire bull terrier, toy and other breeds.

To prepare for the logistic regression model, the potential relationship or association between the number of vaccinations and the age of the dog was tested. Because of non-normality in the distribution of ages in relation to time and number of vaccinations and heterogeneity of variance, an analysis of variance (ANOVA) could not be applied. Instead, a general linear model using count data (family = poisson) was used to align the ages of dogs with the number of vaccinations. Because dog ages were statistically linked with the number of vaccinations, the number of vaccinations as a risk factor was removed from the logistic regression model and only sex, breed, clinic and age were kept for risk factor analysis.

All the records for which sex and breed were not determined or missing (*n* = 100) were removed from the database, and only 381 dog samples were used in the model. A general linear model (family = binomial) with a full model encompassing all risk factors and all possible interactions was initially performed. Using a backward stepwise model selection based on the Akaike information criterion (AIC), the best model was kept (with the smallest AIC). A likelihood ratio test was performed to test the overall significance of each risk factor (multilevel comparison). Because age was analysed as a continuous variable, odds ratio (OR) and prevalence predictions were not calculated for this risk factor. In terms of levels within each categorical risk factor, for comparison purposes, the risk factor associated with the lowest *Campylobacter* spp. prevalence was used as a reference. Adjusted OR that took into account all cofounder variables were calculated, and confidence intervals (CIs). Odds ratio significance was computed using the Wald’s test. To predict the prevalence of *Campylobacter* spp. within different categories for each risk factor, the ‘predict’ function was applied on logistic regression results. Finally, risk factors for *C. jejuni, C. upsaliensis* and *C. coli* were tested using the same approach that was applied for *Campylobacter* spp. risk factor analysis. For all analyses, *p*-values < 0.05 were considered significant.

## Results

### Descriptive analysis

Of the 481 dogs that were tested for *Campylobacter* spp. (see Table 1), 41.58% (95% CI, 37.39% – 46.04%) were positive for *Campylobacter* spp. The distribution of individual *Campylobacter* species was as follows: *C. jejuni* (29.31%; 95% CI, 25.42% – 33.54%) was the most frequent species, followed by *C. upsaliensis* (13.10%; 95% CI, 10.37% – 16.42%) and *C. coli* (5.41%; 95% CI, 3.71% – 7.82%) (see Table 1). Similarly, 6.23% (95% CI, 4.40% – 8.78%) of dogs with mixed infections were also detected: *C. jejuni* + *C upsaliensis*, 3.74% (95% CI, 2.37% – 5.86%) and *C. jejuni* + *C. coli*, 2.49% (95% CI, 1.42% – 4.34%).

**TABLE 1:** Prevalence and risk factors of *Campylobacter* spp. according to clinic, breed, sex and age.

Risk factors†	Adjusted odds ratio (95% CI)	Wald *p*-value	Predicted prevalence (%)	UL‡	LL§
**Clinic**					
Reference – A (*n* = 95)	-	-	16.30	26.38	5.93
B (*n* = 95)	9.61 (4.68–19.75)	< 0.001	65.26	79.24	51.29
C (*n* = 169)	5.68 (2.96–10.88)	< 0.001	54.22	67.74	40.69
D (*n* = 22)	12.23 (4.00–37.36)	< 0.001	72.73	93.89	51.57
**Breed**					
Reference – crossbreed (*n* = 132)	-	-	38.28	50.43	26.13
Moloss (*n* = 29)	Not significant	0.101	58.62	77.48	39.77
**Other (*n* = 9)**	Not significant	0.281	55.56	86.41	24.70
Staff (*n* = 178)	1.73 (1.04–2.88)	0.035	55.11	66.88	43.35
Terrier (*n* = 29)	Not significant	0.125	51.72	70.45	33.00
Toy (*n* = 4)	Not significant	0.982	Not computed – low sample number	-	-
**Sex**					
Reference – female			51.94	64.98	38.90
Male	Not significant	0.389	44.97	58.62	31.32
Age (days)*	-	0.011	-	-	-

UL, upper limit; LL, lower limit; CI, confidence interval.

* , Age was analysed as a continuous variable; no odds ratio or predicted prevalence were calculated.

† , *N* = 381.

‡ , 97.5%.

§ , 0.025%.

### Risk factors

A total of 381 dogs were included in the final model. Logistic regression showed that clinic, age and breed were significant risk factors for carrying *Campylobacter* spp. (see Table 1). No interactions between any of these risk factors were statistically significant in the model. Overall, visiting a particular clinic and the age of the dog were significant risk factors for carrying *C. jejuni* while age was the only risk factor associated with carrying *C. upsaliensis*. Dogs visiting clinics B, C and D were respectively 9.61, 5.68 and 12.3 times more likely to carry *Campylobacter* spp. in comparison to dogs visiting clinic A (reference clinic) (see Table 1). In addition, breed was a predictor of *Campylobacter* spp., with the odds of carrying *Campylobacter* spp. significantly higher in the Staffordshire bull terrier breed in comparison to crossbreed dogs, which had the lowest prevalence of *Campylobacter* spp. Furthermore, the prevalence of *Campylobacter* spp., *C. jejuni* and *C. upsaliensis* increased significantly as dogs grew older.

## Discussion

To our knowledge, this is the first study reporting on the occurrence of *Campylobacter* spp. in dogs visiting rural community veterinary clinics and the risk factors associated with *Campylobacter* spp. carriage in dogs in South Africa. Our results showed that 41.5% of dogs carried *Campylobacter* spp., in line with similar studies, which have reported *Campylobacter* spp. prevalence in dogs ranging from 35% to 43% in Europe and North America (Acke et al. 2009; Holmberg et al. 2015; Parsons et al. 2010; Procter et al. 2014; Workman et al. 2005). However, higher prevalence rates of *Campylobacter* spp. of up to 75.5% have been reported in dogs in different countries including Sweden, Canada, the UK and USA (Chaban et al. 2010; Engvall et al. 2003; Leahy et al. 2017; Parsons et al. 2011).

Variations in *Campylobacter* spp. occurrence rates in dogs have been ascribed to a number of factors, including dog living conditions – whether a dog is confined in a house, a shelter or kennel, or is a stray. Higher *Campylobacter* spp. occurrence rates have been reported in dogs living in shelters or kennels and stray dogs (Baker, Barton & Lanser 1999; Parsons et al. 2011; Procter et al. 2014; Tsai et al. 2007; Workman et al. 2005) in comparison to in-house dogs. Additional factors such as *Campylobacter* spp. culture conditions, including the incubation temperature and atmosphere, as well as antimicrobial supplements that are used for selection of *Campylobacter* spp. in various recovery media or enrichment broths, may also influence *Campylobacter* detection rates (Allos & Lastovica 2008; Aspinall et al. 1996; Lastovica & Le Roux 2001).

*Campylobacter jejuni* was the most frequent *Campylobacter* species in dogs, followed by *C. upsaliensis* and *C. coli* to a lesser extent. This finding is in agreement with previous studies, which have reported *C. jejuni* as the most frequent species in dogs compared to other *Campylobacter* species (Carbonero et al. 2012; Giacomelli et al. 2015; Tsai et al. 2007). However, a number of reports have also found *C. upsaliensis* to be the most frequent species in dogs (Acke et al. 2009; Chaban et al. 2009; Holmberg et al. 2015; Parsons et al. 2011; Rossi et al. 2008). A number of studies have found that *C. upsaliensis* was more frequent in dogs confined in household compounds while *C. jejuni* was more common in stray dogs and shelter or kennel dogs (Carbonero et al. 2012; Leonard et al. 2011; Parsons et al. 2011; Procter et al. 2014). The majority of dog owners in this study indicated that their dogs were not housed in fenced yards and dogs were allowed to leave their living premises and freely roam in the neighbourhood, thereby living a ‘semi-stray’ life. The stray nature of dogs sampled in this study may have played a role in the predominance of *C. jejuni* over *C. upsaliensis*. Roaming, scavenging and hunting behaviours of dogs living a semi-stray life exposes dogs to environments, food and water sources that may favour higher environmental contamination levels with *C. jejuni* compared to *C. upsaliensis*, which has been found to be more frequent in dogs living in-house that are fed home-cooked food (Leonard et al. 2011).

While the role played by *C. jejuni* in human disease is well recognised globally, of particular interest in this study was the presence of dogs infected with *C. upsaliensis. Campylobacter upsaliensis* has emerged in the last 20 years (Bourke et al. 1998) as an important species in dogs worldwide (Chaban et al. 2010; Engvall et al. 2003; Parsons et al. 2010) and a cause of campylobacteriosis in humans (Allos & Lastovica 2008; Couturier et al. 2012; Labarca et al. 2002; Nakamura et al. 2015). This is the first time *C. upsaliensis* has been reported in dogs in South Africa. This finding is of public health significance as *C. upsaliensis* has been previously reported as the third most frequent *Campylobacter* species in South Africa over a period of 10 years in paediatric patients, accounting for 23% of *Campylobacter* spp. cases (Lastovica & Engel 2001).

Carriage of more than one *Campylobacter* species was observed in 6.2% of dogs. Dogs with mixed infections carried *C. jejuni* and *C. upsaliensis* or *C. jejuni* and *C. coli*. Dogs carrying multiple *Campylobacter* species may have been exposed to environments and sources that allow these *Campylobacter* species to thrive favourably. Similar findings have been reported elsewhere (Bojanić et al. 2017; Chaban et al. 2010; Engvall et al. 2003; Hald et al. 2004; Koene et al. 2004). A number of studies have recommended the use of more than one *Campylobacter* spp. culture medium to facilitate the isolation and increase the chance of recovering multiple *Campylobacter* species of public health importance from faecal samples (Endtz et al. 1991; Baker et al. 1999; Koene et al. 2004). In the aforementioned studies in which multiple *Campylobacter* species were detected in individual dogs, at least two media were used to isolate *Campylobacter* spp. While evaluation of the sensitivity of the medium used in this study to recover *Campylobacter* spp. is beyond the scope of this investigation, detection of dogs carrying multiple *Campylobacter* species indicates that Campy CVA agar was a reliable single medium for direct and simultaneous recovery of more than one *Campylobacter* species from dog faeces.

Concerning the different risk factors that were investigated in this study, our findings showed that the overall prevalence of *Campylobacter* spp. and particularly the prevalence of *C. jejuni* and *C. upsaliensis* increased as dogs grew older, with predominance of *C. jejuni* in dogs younger than 1 year in comparison to dogs older than 1 year. Similar studies have reported that dogs less than 1 year old were more likely to be colonised by *Campylobacter* spp. (Acke et al. 2009; Guest, Stephen & Price 2007; Hald et al. 2004; Leahy et al. 2017; Parsons et al. 2010; Procter et al. 2014; Sandberg et al. 2002). The high prevalence of *Campylobacter* spp. in younger dogs may be most probably ascribed to an immature immune system and an underdeveloped enteric microbiota that is unable to outcompete and displace *Campylobacter* spp. in the intestine. This finding was not surprising as the dog population under study was skewed towards a higher number of dogs younger than 1 year (88%) compared to dogs that were older than 1 year.

Consistent with previous studies, the prevalence of *Campylobacter* spp. was not significantly different in male and female dogs (Nair et al. 1985; Olson & Sandstedt 1987; Sandberg et al. 2002; Torre & Tello 1993). However, breed was overall a predictor for *Campylobacter* carriage, with the Staffordshire bull terrier breed more likely to carry *Campylobacter* spp. in comparison to crossbreed dogs. Although breed has never been reported as a risk factor for *Campylobacter* spp. occurrence in dogs, this finding may indicate that dogs belonging to the Staffy breed, which is a pure breed, may be more susceptible to disease in comparison to crossbreeds, which are generally considered more resistant to disease.

Visiting a particular clinic was identified as a risk factor for carrying *C. jejuni*, with dogs visiting clinics B, C and D presenting a higher risk of carrying *Campylobacter* spp. and particularly *C. jejuni*. While the reasons behind this finding are not clear, the authors postulate that there are yet-unidentified factors such as dog living conditions (in-house vs. stray dogs) that may be favouring a higher occurrence rate of *C. jejuni* in dogs living in the communities serviced by clinics B, C and D in comparison to clinic A, which had the lowest *Campylobacter* spp. prevalence.

## Conclusion

This study provides useful information on the prevalence and risk factors of *C. jejuni, C. upsaliensis* and *C. coli* in dogs visiting rural community veterinary clinics in South Africa. Our results indicate that dogs visiting the veterinary rural community clinics under study are reservoirs and may be an important source of *Campylobacter* spp. for humans. However, a limitation of this study is that the dogs studied were not recruited randomly and the prevalence of *Campylobacter* presented in this study may not be a reflection of the larger dog population of South Africa. Future epidemiological and characterisation studies comparing dog and human *Campylobacter* spp. isolates are needed to establish the zoonotic potential of *Campylobacter* spp. carried by dogs in South Africa.
